# Living-donor lobar lung transplantation for pulmonary Langerhans cell histiocytosis complicated by extensive thrombi in central pulmonary arteries

**DOI:** 10.1186/s40792-024-01968-w

**Published:** 2024-07-11

**Authors:** Naoki Date, Akihiro Ohsumi, Kenji Minatoya, Hiroshi Date

**Affiliations:** 1 Department of Thoracic Surgery, Kyoto University Hospital, 54 Shogoin Kawahara-Cho, Sakyo-Ku, Kyoto, 606-8507 Japan; 2 Department of Cardiovascular Surgery, Kyoto University Hospital, 54 Shogoin Kawahara-Cho, Sakyo-Ku, Kyoto, 606-8507 Japan

**Keywords:** Pulmonary Langerhans cell histiocytosis, Lung transplantation, Thrombosis, Pulmonary hypertension

## Abstract

**Background:**

Pulmonary Langerhans cell histiocytosis (PLCH) is a rare disorder characterized by the proliferation of Langerhans cells along the small airways, which causes nodular and cystic changes in the lung parenchyma. Lung transplantation can be a life-saving option for patients with severe respiratory failure or pulmonary hypertension. Herein, we present a case of successful lung transplantation in a patient with PLCH who developed unusually large thrombi in the central pulmonary artery.

**Case presentation:**

A 47-year-old woman with 16-year history of PLCH with rapidly developing respiratory failure was admitted to our hospital for the evaluation of a lung transplant. Enhanced computed tomography revealed large thrombi in dilated central pulmonary arteries. Right heart catheterization revealed severe pulmonary hypertension, with a mean pulmonary artery pressure of 48 mmHg. The thrombi shrank markedly after 3 months of anticoagulation therapy. However, the respiratory status of the patient did not improve. We performed bilateral living-donor lobar lung transplantation with thrombectomy under extracorporeal membrane oxygenation for the remaining thrombi in the main pulmonary arteries. The dilated main pulmonary arteries of the recipient required direct plication for size mismatch. The patient survived in good condition for more than 2 years with no recurrence of thrombosis.

**Conclusion:**

Preoperative anticoagulation therapy for massive thrombi in the pulmonary arteries was effective and led to safe lung transplantation.

## Background

Pulmonary Langerhans cell histiocytosis (PLCH) is a rare lung disease predominantly occurring in young adults. The proliferation of Langerhans cells along the small airways causes nodular and cystic changes in the lung parenchyma, which can lead to respiratory failure and severe pulmonary hypertension (PH) [1]. Lung transplantation (LT) is a therapeutic option for end-stage PLCH, with outcomes comparable to those of other lung diseases [2].

In this case report, we describe a patient with PLCH who rapidly developed respiratory failure with large thrombi in the central pulmonary arteries and underwent living-donor lobar lung transplantation (LDLLT) with thrombectomy and adjustment of the size discrepancy in the pulmonary arteries. Although the prevalence of pulmonary embolism is reported to be 11–15 times higher in patients with chronic obstructive pulmonary disease and interstitial lung disease than in the general population [3], no reports have described lung transplantation for end-stage pulmonary disease with massive pulmonary thrombosis.

## Case presentation

A 47-year-old woman with a history of smoking was diagnosed with PLCH through a transbronchial lung biopsy. Despite smoking cessation, cystic changes in the lungs progressed, and the patient developed pneumothorax and central diabetes insipidus owing to the extrapulmonary involvement of PLCH, which required desmopressin. At the age of 36 years, the patient was referred to our hospital for lung transplant evaluation; however, it was deemed premature for transplantation given her stable respiratory status and preserved exercise tolerance at that time. Subsequently, the patient's condition gradually deteriorated. She experienced repeated pneumothorax and became oxygen inhalation dependent at the age of 39 years. The patient refused a second lung transplant evaluation because of financial problems; therefore, conservative treatment was continued for several years.

**Fig. 1:**
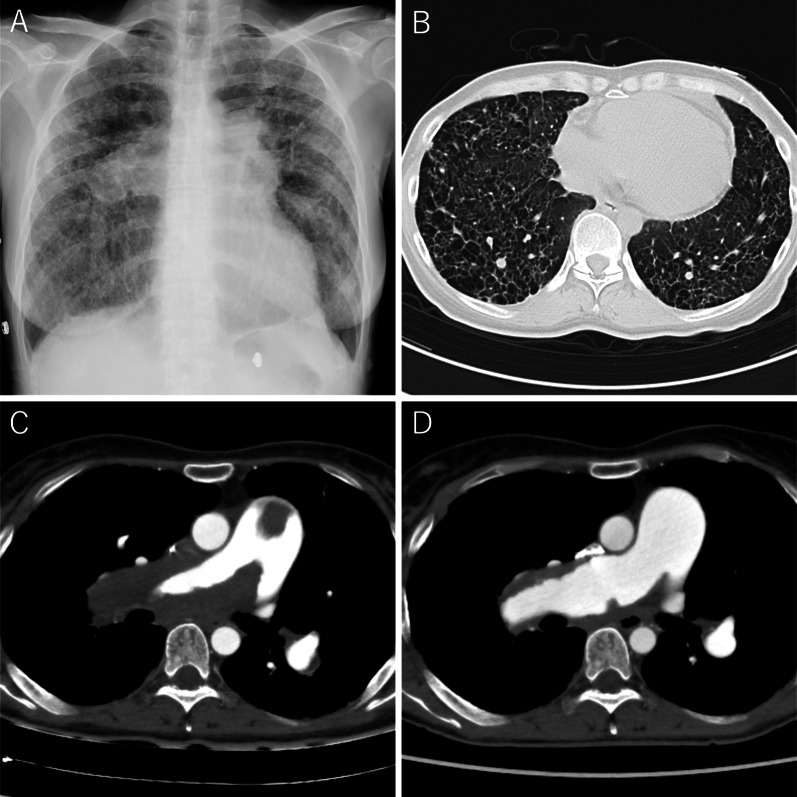
Radiological images. **A** Bilateral enlarged pulmonary artery shadows are seen in the chest radiograph. **B** The computed tomography shows numerous thin-walled pulmonary cysts. **C** Massive thrombi in dilated pulmonary artery extending from the pulmonary trunk to both main pulmonary arteries are revealed at initial enhanced computed tomography. **D** Follow-up computed tomography after 3 months of anticoagulation therapy reveals markedly shrinking thrombi limited in the main pulmonary artery wall

At the age of 47, the patient rapidly developed leg edema and exertional dyspnea. She was referred to a local hospital, and ultrasonic cardiography suggested PH. Her respiratory status did not improve despite diuretic therapy. Consequently, she was admitted to our hospital because of severe respiratory failure. The peripheral oxygen saturation was 92% with 10 L/min oxygen inhalation. Chest radiography revealed bilateral reticular opacities and enlarged pulmonary arterial shadows. Contrast-enhanced computed tomography (CT) revealed numerous thin- and thick-walled pulmonary cysts, as well as large thrombi extending from the pulmonary trunk to both the main pulmonary arteries without deep venous thrombosis and peripheral pulmonary artery thrombosis (Fig. 1A–C). Right heart catheterization revealed PH with a mean pulmonary artery pressure of 48 mmHg. The patient was treated with riociguat and macitentan for PH along with systemic anticoagulation with warfarin. The prothrombin time-international normalized ratio (PT-INR) was maintained between 2.5 and 3.0. After 3 months of anticoagulation therapy, the thrombi disappeared from the pulmonary trunk and were limited to both main pulmonary arteries on CT (Fig. 1D). However, the patient remained bedridden owing to severe exertional dyspnea and was easily desaturated even under nasal high-flow oxygen therapy. The patient was evaluated and deemed a candidate for LT. However, it was difficult to wait for deceased-donor LT, therefore we decided to perform LDLLT. Her brother and daughter were suitable donors, and the right lower lobe of her brother and the left lower lobe of her daughter were estimated to provide 77% of the recipient’s predicted forced vital capacity.

### Operation

**Fig. 2:**
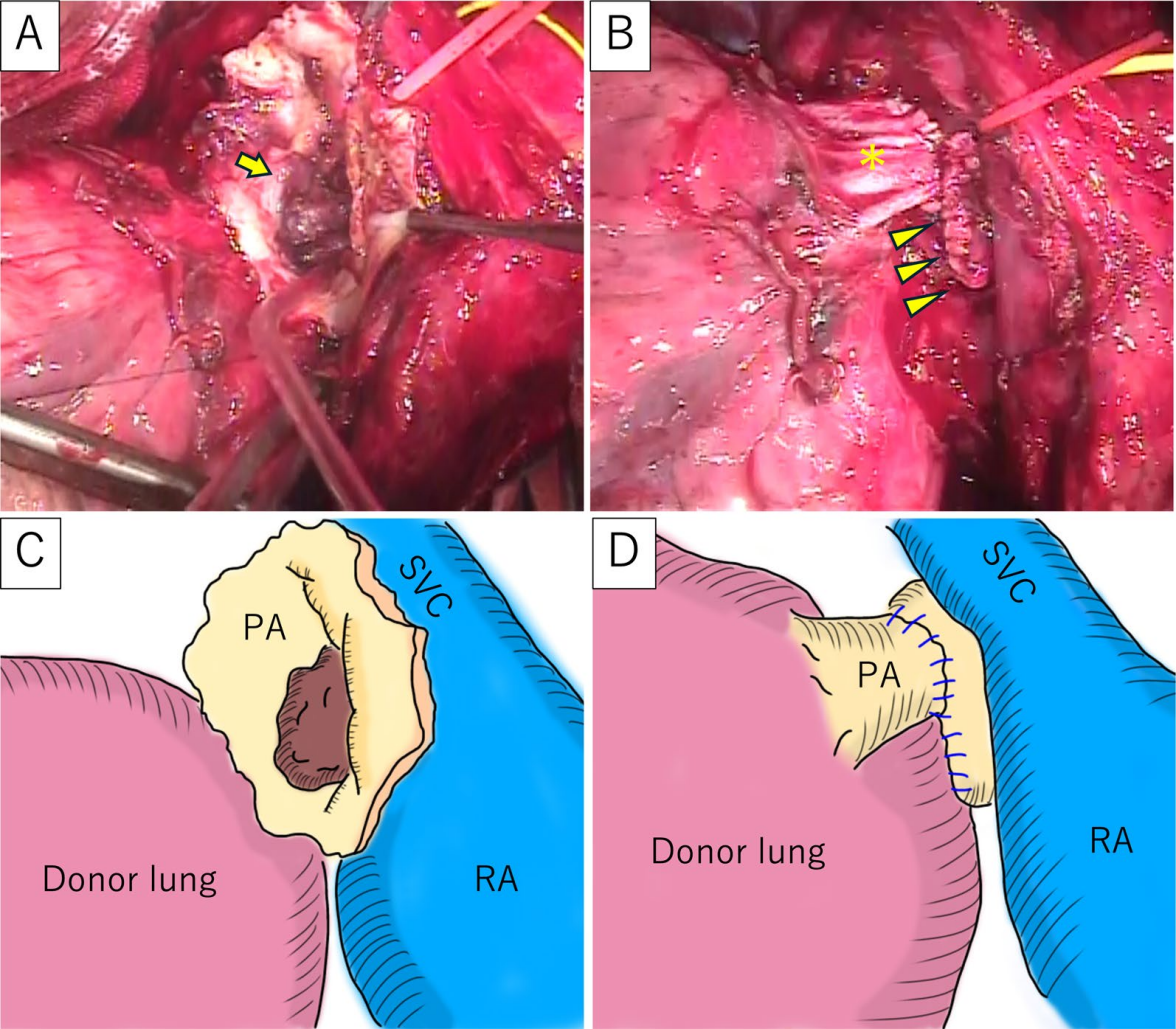
Intraoperative findings. **A** Thrombi (arrow) were observed inside the opened right main pulmonary arteries. **B** The pulmonary artery of the graft (asterisk) was anastomosed to the dilated main pulmonary artery of the recipient, the size of which was reduced with direct plication (arrowheads). **C** The schema of **A**. **D** The schema of Fig. 2B. SVC: superior vena cava, PA: pulmonary artery, RA: right atrium

The surgery was performed through a clamshell incision. Central extracorporeal membrane oxygenation (ECMO) was established via arterial cannulation of the ascending aorta and venous cannulation from the right atrium to the superior vena cava and right femoral vein. The right lung was removed using staplers for vascular and bronchial division. The right main pulmonary artery was encircled between the superior vena cava and the ascending aorta for subsequent clamping at the proximal of the thrombi. The donor’s right lower lobe was implanted, and the graft’s lower bronchus and pulmonary vein were anastomosed to the main bronchus and left superior pulmonary vein of the recipient, respectively. The right main pulmonary artery was clamped between the superior vena cava and the ascending aorta. The remaining thrombi in the right main pulmonary artery were removed (Fig. 2A). To deal with the size mismatch between the dilated recipient’s right main pulmonary artery and the lobar pulmonary artery of the graft, the recipient’s pulmonary artery was wedge-excised and plicated. Anastomosis of the pulmonary arteries was conducted in an end-to-end fashion (Fig. 2B), and the right lung was reventilated and reperfused. The left lower lobe of the patient’s daughter was transplanted using a similar technique. The ECMO was smoothly weaned off, and the ECMO circuit was removed. The PaO2 on admission to the intensive care unit was 498 mmHg with 100% oxygen inhalation.

### Postoperative course

Anticoagulation therapy with heparin (10,000 U/day) was initiated on postoperative day two. Epidural anesthesia was not used because of the need for anticoagulation therapy. One week after transplantation, acute rejection was suspected and steroid pulse therapy was administered. A human leukocyte antigen (HLA) antibody test was concurrently performed and revealed positivity for donor-specific antibodies against HLA class II antigens in her daughter. The patient was diagnosed with antibody-mediated rejection and treated with plasmapheresis for 5 days followed by intravenous immunoglobulin therapy for 3 days. Subsequently, anti-thymocyte globulin therapy was also given for 5 days considering the prolongation of clinical symptoms, and the patient’s respiratory status gradually improved following treatment. A CT scan conducted 1 month after the operation showed no residual thrombi in the pulmonary arteries. Consequently, the anticoagulation therapy was switched to warfarin, with the PT-INR maintained between 1.5 and 2.5, as required for the treatment of pulmonary arterial hypertension and chronic thromboembolic pulmonary hypertension. The patient was discharged on postoperative day 64 without any oxygen requirement. At the two-year follow-up, she was doing well and was leading a normal life.

## Discussion

PLCH is a part of rare neoplastic disorder characterized by monoclonal proliferation and infiltration of organs by Langerhans cells accompanied by a strong inflammatory response [4]. The lungs may be involved as isolated organs or as part of a multi-organ disease. Common extrapulmonary involvements are bones, skin, and pituitary gland [4, 5]. Our patient had central diabetes insipidus secondary to pituitary involvement. PLCH is known to be strongly associated with smoking, and more than 90 percent of patients with this disease have a smoking history. Smoking cessation is an essential treatment that leads to partial regression and subsequent stabilization in most patients. The efficacy of other treatments, including corticosteroids and chemotherapeutic agents, is limited and not well studied [1, 4]. Although patients with PLCH have shorter long-term survival than the general population, the percentage of patients progressing to need LT is thought to be very small (< 1%) [2, 4]. LT is a salvage therapeutic option for patients with end-stage PLCH. The reported post-transplant 5-year survival rate is approximately 50%, which is comparable to that of patients with other lung diseases [2].

Pulmonary hypertension is frequently observed in patients with PLCH. The reported prevalence of PH is 41% in patients with PLCH and 92% in those with PLCH requiring LT [4, 6]. Our patient exhibited severe PH with dilated pulmonary arteries that required adjustment for size mismatch. Among the various techniques for anastomosis of pulmonary arteries with a size mismatch, including tuck suture or oblique stapling [7], we applied a direct plication method considering the location of the orifices and thickening of the vessel wall due to severe inflammation. Our findings indicate that careful hemodynamic assessment is required for patients with end-stage PLCH.

In this case, the patient developed massive thrombi in the pulmonary arteries. Although there have been no reports on the association between PLCH and thrombosis, Moser et al. reported three cases of extensive central thrombi in patients with primary PH and suggested that the thrombi were engrafted upon intimal injury induced by long-standing PH [8]. Considering our patient’s severe PH associated with PLCH, we assumed that the combination of the inflammatory response of Langerhans cells and endothelial damage in the dilated pulmonary arteries might contribute to thrombus formation. Additionally, the patient had central diabetes insipidus, potentially causing a dehydration-induced hypercoagulable state that could accelerate thrombosis [9]. We initially assumed that cardiopulmonary bypass was necessary to perform a thrombectomy of the pulmonary trunk immediately after moving to our hospital. However, the thrombi markedly responded to preoperative anticoagulation therapy, and the remaining thrombi were successfully removed without endarterectomy under ECMO by clamping the right main pulmonary artery between the ascending aorta and the superior vena cava. These findings suggest an acute formation of thrombi, distinct from the chronic thromboembolic pulmonary hypertension. The thrombolysis makes it possible to perform transplantation without cardiac arrest using cardiopulmonary support, leading to a reduced risk of perioperative bleeding and enabling the immediate and safe initiation of postoperative anticoagulation therapy. To the best of our knowledge, this is the first documented case of lung transplantation combined with concurrent thrombectomy in a patient with end-stage lung disease.

## Conclusions

Preoperative anticoagulation therapy for massive thrombi in the pulmonary artery may be effective and lead to safe lung transplantation with concurrent thrombectomy.

## Data Availability

None.

## Abbreviations

PLCH: Pulmonary Langerhans cell histiocytosis
PH: Pulmonary hypertension
LT: Lung transplantation
LDLLT: Living-donor lobar lung transplantation
CT: Computed tomography
PT-INR: Prothrombin time-international normalized ratio
ECMO: Extracorporeal membrane oxygenation
HLA: Human leukocyte antigen
